# Impact of New Inflammation/Nutrition-Based Indicators on Prognosis in Elderly Patients With Colorectal Cancer

**DOI:** 10.1155/mi/7843467

**Published:** 2025-11-19

**Authors:** Wenda Xu, Chen Qin, Hanyu Yang, Haoyu Cui, Shuo Liu, Zechen Lu, Wenchang Yang, Jilin Hu

**Affiliations:** ^1^Department of Gastrointestinal Surgery, The Affiliated Hospital of Qingdao University, Qingdao, China; ^2^Department of Medical Oncology, Qingdao Central Hospital, University of Health and Rehabilitation Sciences, Qingdao, China

**Keywords:** colorectal cancer, modified cachexia index, nomogram, prognosis

## Abstract

**Objective:**

This study aimed to investigate the impact of new inflammation/nutrition-based indicators—the modified cachexia index (mCXI) on the prognosis of elderly patients with colorectal cancer and develop a nomogram model.

**Methods:**

A retrospective analysis was conducted using clinical data from patients over 70 years old diagnosed with colorectal cancer, at the Affiliated Hospital of Qingdao University between July 2018 and December 2023. Univariate and multivariate Cox regression analyses, based on overall survival (OS) and recurrence-free survival (RFS), identified independent prognostic factors. A nomogram prediction model was constructed using multivariate analysis.

**Results:**

The study included 456 patients, comprising 273 males (59.9%) and 183 females (40.1%), with a mean age of 75 years. The median follow-up was 41 months. The 1-, 3-, and 5-year OS rates were 85%, 65%, and 50%, respectively, while the 1-, 3-, and 5-year RFS rates were 80%, 55%, and 40%, respectively. Multivariate Cox regression identified age, tumor T stage, tumor N stage, and mCXI as independent factors affecting both OS and RFS. A nomogram prognostic model was developed, with area under the ROC curve (AUC) values of 0.742, 0.809, and 0.799 for 1-, 3-, and 5-year OS, respectively, and AUC values of 0.769, 0.798, and 0.797 for 1-, 3-, and 5-year RFS, respectively. Calibration curves indicated strong agreement with the ideal model and decision curve analysis confirmed the model's robust predictive accuracy.

**Conclusion:**

The mCXI serves as an independent risk factor for both RFS and OS in elderly patients with colorectal cancer. The nomogram prediction model demonstrates strong prognostic value for this patient group.

## 1. Introduction

Colorectal cancer (CRC) ranks the third most prevalent malignancy and the second leading cause of cancer-related deaths globally [1]. In China, CRC ranks among the top five cancers in terms of incidence and is the fourth leading cause of cancer-related mortality [2]. Surgical resection remains the primary treatment for Stages I and II CRC [3]. However, many patients do not present with typical clinical symptoms in the early stages and often seek medical attention only when the disease has progressed to later stages, missing the optimal window for surgical intervention. For these patients, preoperative chemoradiotherapy followed by surgery is the standard approach. Despite advancements in surgical techniques and perioperative care, prognosis improvements particularly for elderly patients have been limited. Besides, demographic shifts toward an aging society have resulted in a growing prevalence of CRC in elderly patient populations [4]. Research indicates that elderly patients with CRC generally have poorer prognoses than their younger counterparts [5], highlighting the importance of prognostic evaluation to guide treatment decisions in this group.

The International Union Against Cancer's tumor node metastasis (TNM) classification is widely used to guide CRC treatment. However, patients with the same pathological stage can exhibit varying prognoses. Increasing evidence suggests that nutritional status significantly correlates with clinical outcomes in CRC patients, particularly among the elderly [6]. A study has shown that nutritional status scores correlate with tumor progression and poor outcomes in patients with CRC undergoing curative resection [7]. Additionally, Xie et al. [8] established CT-quantified sarcopenia as a valid prognostic indicator for survival trajectories in geriatric CRC cases. Contemporary research demonstrates the cachexia index (CXI) has been identified as a useful predictor of cancer cachexia and prognosis in cancer individuals [9, 10]. The CXI calculation requires the skeletal muscle index (SMI), which is derived from CT scans. Given the time-consuming nature of image annotation and the challenges in accurately identifying the position and size of tissues, researchers have sought alternative biomarkers. Yuan et al. [11] developed and validated a modified CXI (mCXI), calculated using serum albumin (ALB), the neutrophil–lymphocyte ratio (NLR), and the urea–creatinine ratio (UCR), to predict the prognosis of patients with CRC after surgery.

This study employed mCXI to predict RFS and OS in elderly patients with CRC and developed predictive models integrating mCXI with TNM classification, aiming to enhance postoperative prognostication for this patient group.

## 2. Materials and Methods

### 2.1. General Information

**Figure 1:**
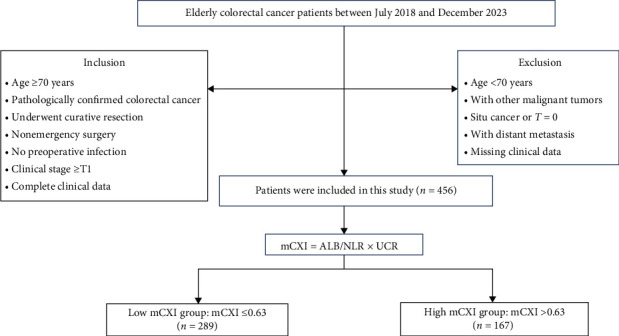
Study population selection flowchart.

Clinical data from patients with CRC aged over 70 years, treated at the Affiliated Hospital of Qingdao University between July 2018 and December 2023, were retrospectively collected. The inclusion criteria were (1) age ≥70 years, (2) pathologically confirmed CRC, (3) underwent curative resection, (4) nonemergency surgery, (5) no preoperative infection, (6) clinical stage ≥ T1, and (7) complete clinical data. Exclusion criteria included (1) presence of other malignancies, (2) preoperative distant metastasis, (3) tumor T stage of 0 or carcinoma in situ, and (4) incomplete clinical data. The study was approved by the Medical Ethics Committee of the Affiliated Hospital of Qingdao University and informed consent was waived. A detailed screening process is provided in the flowchart (Figure 1).

### 2.2. Observation Indicators

A total of 11 clinicopathological indicators were analyzed: mCXI, age, gender, SMI, carcinoembryonic antigen (CEA) level, tumor T stage, tumor N stage, Ki-67 index, presence of neural invasion, presence of vascular invasion, and neoadjuvant therapy. The CXI, a significant prognostic marker for cancer individuals, is calculated as the product of SMI and serum ALB, divided by the NLR (CXI = (ALB × SMI)/NLR). The mCXI is derived by adapting the CXI, using the ratio of preoperative serum ALB to the product of NLR and the urea-to-creatinine ratio (UCR; mCXI = ALB/(NLR × UCR). The cutoff of mCXI was determined based on recurrence-free survival (RFS) data, using the highest specificity and sensitivity (maximal Youden index). SMI is determined as the total skeletal muscle area (cm^2^) at the third lumbar vertebra (L3) level on computed tomography (CT) scans, divided by the square of height (m^2^) [12, 13]. CEA is a widely recognized serum biomarker for CRC detection and monitoring [14]. The CEA was measured by immunoassays in clinical laboratories using blood samples from patients. Ki-67, a nuclear protein associated with cell proliferation, is commonly used to assess proliferative activity and serves as a reliable marker for cell proliferation [15]. The measurement of Ki-67 requires obtaining tumor tissue samples, which are observed and evaluated by pathologists under a microscope. The Ki-67 index (%) is calculated as (number of positive cell nuclei/total number of tumor cell nuclei) multiplied by 100%.

### 2.3. Follow-Up Data

Follow-up occurred every 3 months during the first year, every 6 months during the second year, and annually thereafter. Follow-up methods included telephone calls, letters, WeChat communication, and return visits. Follow-up data included the tumor recurrence, metastasis, or death, along with relevant timelines, biochemical test results, imaging findings, and colonoscopy outcomes. Local recurrence or distant metastasis was confirmed via CT scans, colonoscopy, or pathological examination. The follow-up period continued until December 2023. RFS was defined as the time from initial surgical resection to tumor recurrence, metastasis, or the last follow up. Overall survival (OS) was defined as the time from the initial surgical resection to the end of the follow up or death from any cause.

### 2.4. Statistical Methods

*t*-Tests or Mann–Whitney tests were used for continuous variables and *χ*^2^ test was used for categorical variables. Survival curves were generated using the Kaplan–Meier method to compare survival outcomes across groups. Independent determinants of prognosis were ascertained using stepwise Cox regression analysis and continuous predictors were modeled as continuous variables. Variables with a *p* value < 0.05 were incorporated into the multivariate model. Based on the multivariate analysis, a nomogram prediction model was developed. The model's predictive performance and accuracy were evaluated using ROC curves, calibration curves, and decision curve analysis (DCA). The cutoff values, comparison between two groups, univariate and multivariate cox regression analysis of OS and RFS were calculated using IBM SPSS Statistics for Windows, Version 27.0. The time-dependent ROC curve, calibration curve, decision curve, Delong test, and nomogram were calculated using R language (version 4.4.1). The “pROC” package was used for ROC analyses. A *p* value < 0.05 was considered statistically significant.

## 3. Results

### 3.1. Clinicopathological Characteristics

Between July 2018 and December 2023, 1400 patients with CRC underwent surgical treatment at our hospital, with 456 patients meeting the inclusion and exclusion criteria for this study. The cohort had a mean age of 76.5 ± 5.2 years and a mean SMI of 40.2 ± 5.8 kg/m^2^. Of these patients, 222 (48.7%) were over 75 years old, 273 (59.9%) were male, 152 (33.3%) had a tumor diameter > 5 cm, 225 (49.3%) had an SMI > 40 kg/m^2^, 289 (63.4%) had a CEA level > 3.41 ng/mL, 347 (76.1%) had T3 or T4 staging, 82 (18.0%) had N2 staging, 198 (43.4%) had a Ki-67 index >75%, 180 (39.5%) had neural invasion, 111 (24.3%) had vascular invasion, and 22 (4.8%) received preoperative neoadjuvant therapy. The average CXI was 388 and the average mCXI was 0.61. The range of mCXI values was 0.11–4.02. Patients with mCXI ≤ 0.63 were assigned to the low mCXI group, while those with mCXI > 0.63 were assigned to the high mCXI group. Of the 456 patients, 289 had low mCXI levels and 167 had high mCXI levels. A comparative analysis between the two groups revealed significant differences in age and Ki-67 levels (*p* < 0.05). Notably, no meaningful disparities were observed in SMI, gender, tumor diameter, T stage, N stage, neural invasion, vascular invasion, or preoperative neoadjuvant therapy (*p* > 0.05; Table 1).

### 3.2. Univariate and Multivariate Cox Regression Analysis

#### 3.2.1. Univariate and Multivariate Cox Regression Analysis for OS

Univariate Cox regression analysis identified age, T3 or T4 tumor staging, N2 staging, vascular invasion, and mCXI as significantly associated with poor prognosis in patients with CRC (*p* < 0.05). Multivariate Cox regression analysis further confirmed that age (HR = 1.054, 95% CI: 1.010–1.100, *p*=0.017), T3 or T4 tumor staging (HR = 2.891, 95% CI: 1.232–6.783, *p*=0.015), N2 staging (HR = 4.249, 95% CI: 2.579–7.000, *p* < 0.001), and mCXI (HR = 0.124, 95% CI: 0.042–0.359, *p* < 0.001) were independent risk factors for OS in elderly patients with CRC (*p* < 0.05; Table 2).

#### 3.2.2. Univariate and Multivariate Cox Regression Analysis for RFS

Univariate Cox regression analysis revealed that age, T3 or T4 tumor staging, N2 staging, vascular invasion, and mCXI were significantly linked to poor prognosis in patients with CRC (*p* < 0.05). In the multivariate analysis, age (HR = 1.046, 95% CI: 1.002–1.092, *p*=0.039), T3 or T4 tumor staging (HR = 2.943, 95% CI: 1.256–6.896, *p*=0.013), N2 staging (HR = 4.335, 95% CI: 2.627–7.153, *p* < 0.001), and mCXI (HR = 0.128, 95% CI: 0.045–0.369, *p* < 0.001) emerged as independent risk factors for RFS in elderly patients with CRC (*p* < 0.05; Table 3).

### 3.3. Construction and Evaluation of the Nomogram Prediction Model

The performance of the nomogram model, developed based on age, T staging, N staging, and mCXI levels, in predicting 1-, 3-, and 5-year OS and RFS in elderly patients with CRC was assessed using ROC curves, calibration curves, and DCA.

#### 3.3.1. OS Analysis

**Figure 2:**
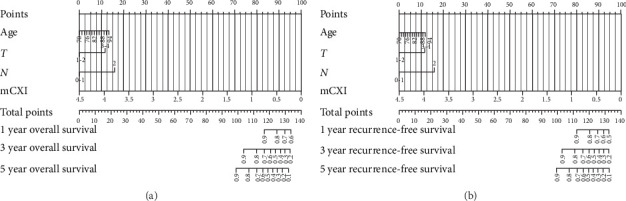
Prognostic nomograms for elderly colorectal cancer patients. (a) Nomogram for predicting 1-, 3-, and 5-year OS. (b) Nomogram for predicting 1-, 3-, and 5-year RFS.

**Figure 3:**
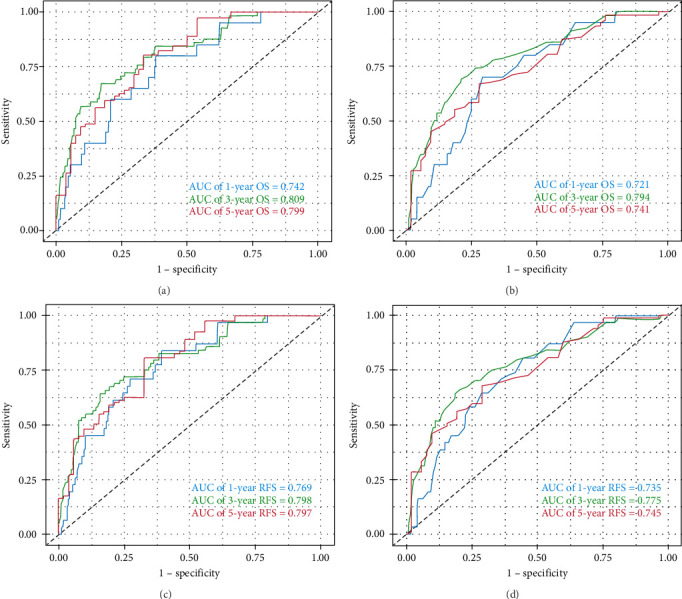
Receiver operating characteristic (ROC) curves for the nomogram model. (a) 1-, 3-, and 5-year OS (with mCXI) prediction. (b) 1-, 3-, and 5-year OS (without mCXI) prediction. (c) 1-, 3-, and 5-year RFS (with mCXI) prediction. (d) 1-, 3-, and 5-year RFS (without mCXI) prediction.

**Figure 4:**
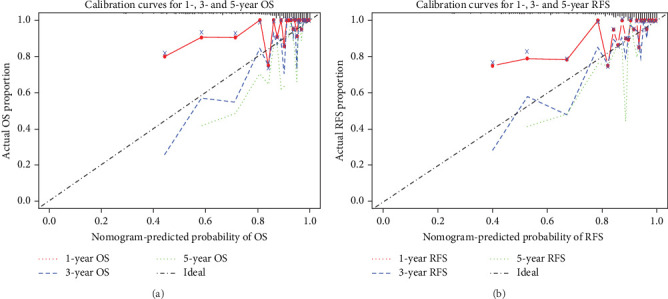
Calibration curves for the nomogram model. (a) 1-, 3-, and 5-year OS prediction. (b) 1-, 3-, and 5-year RFS prediction.

**Figure 5:**
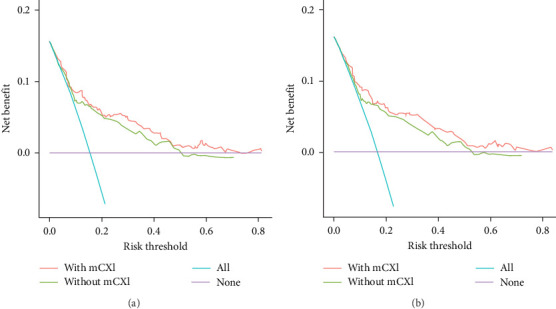
Decision curve analysis (DCA) for the nomogram models with and without mCXI. (a) Decision curve analysis of OS. (b) Decision curve analysis of RFS.

• Nomogram: The nomogram quantified the effect of each variable on survival probability (Figure 2A).
• ROC curve analysis: The AUC values for 1-, 3-, and 5-year OS were 0.742, 0.809, and 0.799, respectively. The highest AUC value (0.809) for 3-year OS indicated the model's optimal performance in predicting 3-year survival (Figure 3A).
• Calibration curves: The calibration curves showed excellent agreement between actual survival rates and predicted survival probabilities for 1-, 3-, and 5-year OS. Data points were closely aligned with the diagonal, confirming the model's high accuracy (Figure 4A).
• DCA: The clinical utility of the nomogram for predicting OS was assessed using DCA.  Figure 5A compares the decision curves of the models with and without mCXI. The results demonstrated that across a wide range of threshold probabilities, the model incorporating mCXI provided a higher net benefit than the model without mCXI, indicating that the inclusion of mCXI enhances the clinical practicality of the model for OS prediction (Figure 5A).

#### 3.3.2. RFS Analysis

• Nomogram: The nomogram also quantified the impact of each variable on RFS probability (Figure 2B).
• ROC curve analysis: The AUC values for 1-, 3-, and 5-year RFS were 0.769, 0.798, and 0.797, respectively, demonstrating strong predictive performance at all time points (Figure 3C). Furthermore, to further clarify the clinical value of mCXI in predicting RFS, we analyzed the AUC of the model without mCXI for predicting prognosis.
• Calibration curves: Like OS, the calibration curves for RFS demonstrated a high degree of agreement between the actual and predicted survival probabilities, with data points closely following the diagonal, validating the model's accuracy for RFS prediction (Figure 4B).
• DCA: Similarly, DCA was performed for the nomogram predicting RFS. As shown in Figure 5B, the decision curve of the model with mCXI was above that of the model without mCXI across most threshold probabilities. This indicates that incorporating mCXI into the predictive model significantly increases its net benefit and decision-support value for RFS prediction (Figure 5B).

### 3.4. The Effect of mCXI in Prognostic Model

• To further clarify the clinical value of mCXI in predicting OS and RFS, we analyzed the AUC of the model without mCXI for predicting prognosis. The results showed that the AUC values for 1-, 3-, and 5-year OS in models (without mCXI) were 0.721, 0.794, and 0.741 (Figure 3B). The Delong test showed that the value of the model incorporating mCXI for predicting 3- and 5-year OS was significantly higher than that of the model without mCXI (Table 4). The results showed that the AUC values for 1-, 3-, and 5-year RFS in models (without mCXI) were 0.735, 0.775, and 0.745 (Figure 3D). The Delong test showed that the value of the model incorporating mCXI for predicting 3- and 5-year RFS was significantly higher than that of the model without mCXI (Table 4). In addition, the DCA curve showed that the inclusion of mCXI increased the clinical utility of nomogram.
• To further evaluate the contribution of mCXI to the predictive ability of the models, we calculated the Akaike information criterion (AIC) values for models with and without mCXI. The results showed that for OS prediction, the AIC value of the model containing mCXI was 792.696, which was substantially lower than that of the model without mCXI (815.868). Similarly, for RFS prediction, the AIC value of the model with mCXI (798.737) was also lower than that of the model without mCXI (821.792). The lower AIC values indicate that incorporating the mCXI variable resulted in a better balance between model complexity and goodness-of-fit, further confirming the value of mCXI in enhancing the model's predictive performance.

### 3.5. The Effect of CXI Vs mCXI in Nomogram

• We investigated whether adding CXI to the model would increase predictive value for the model. The results showed that the AUC values for 1-, 3-, and 5-year OS in models (with CXI) were 0.725, 0.787, and 0.752 (Supporting Information 1: Figure S1 and Supporting Information 2: Table S1), which did not improve the predictive value of the model in OS. These results further demonstrated a model with CXI instead of mCXI resulted in lower predictive ability.
• Same as OS, we investigated whether adding CXI to the model would increase predictive value of RFS for the model. The results showed that the AUC values for 1-, 3-, and 5-year RFS in models (with CXI) were 0.736, 0.771, and 0.754 (Supporting Information 1: Figure S1 and Supporting Information 2: Table S1), which revealed that a model with CXI instead of mCXI resulted in lower predictive ability.

## 4. Discussion

CRC ranks as the third most prevalent cancer globally and is the second leading cause of cancer-related mortality, presenting substantial challenges in the prognosis management of elderly patients. While advancements in surgical techniques and comprehensive treatment strategies have significantly improved survival rates, postoperative recurrence and metastasis continue to be major clinical hurdles. This study was the first to explore the role of the mCXI in the prognostic evaluation of elderly patients with CRC and to develop a nomogram prediction model based on mCXI. The findings demonstrate that mCXI is an independent risk factor for both RFS and OS in elderly patients with CRC. Furthermore, the mCXI-based nomogram model exhibits excellent accuracy and clinical utility. These results provide a novel theoretical foundation for individualized treatment strategies and prognostic assessment in elderly patients with CRC.

The prognosis of CRC is influenced by numerous factors. Univariate and multivariate Cox regression analyses in this study identified age, T staging, N staging, and mCXI as independent risk factors affecting both OS and RFS in elderly patients with CRC. The TNM staging system has long been considered the gold standard for evaluating CRC prognosis, and advanced age is widely acknowledged as a critical factor influencing cancer outcomes [16]. The findings of this study align with previous research, further underscoring the critical role of mCXI in prognostic assessment for elderly patients with CRC.

SMI determined by CT is a valuable indicator for assessing skeletal muscle mass, widely used in clinical studies examining sarcopenia [8, 17, 18]. Sarcopenia is frequently observed in elderly patients with cancers and results from a negative balance between insufficient food intake and heightened tumor metabolism. The SMI, a widely used method for measuring muscle atrophy, has been significantly associated with survival outcomes in elderly populations and cancer individuals [19]. The SMI has also been identified as a useful prognostic marker for patients with CRC [20, 21]. However, in this study, sarcopenia as measured by SMI was not found to be an independent risk factor, potentially due to biases in the SMI measurement process. While preoperative CT scans are routinely performed in patients with CRC, determining SMI requires abdominal CT scans at the level of the L3 vertebra [22, 23]. The complexity and substantial workload involved in the measurement process have limited the clinical applicability of SMI. Consequently, this study aimed to identify alternative, more accessible indicators for prognostic assessment.

Cancer cachexia affects approximately 50% of patients with CRC and contributes to 20% of cancer-related deaths [24]. Therefore, we explored the predictive value of another marker-CXI on prognosis. However, our study indicated that CXI was not an independent risk factor affecting prognosis, which may be related to the fact that CXI is calculated by SMI, limiting its broader clinical application. Furthermore, our study revealed that adding CXI to the model did not improve the predictive value of RFS and OS. To address this, the study examined the mCXI, which, like CXI, offers a comprehensive evaluation of malignancies. The mCXI incorporates serum ALB, NLR, and UCR. The difference between mCXI and CXI is that mCXI replaces SMI with UCR, avoiding CXI's dependence on CT imaging (SMI) and instead using more easily obtainable serum markers (UCR). Research has shown that UCR is inversely correlated with muscle cross-sectional area [25], and elevated UCR levels are associated with muscle bioenergetic failure [26], muscle catabolism [27], and persistent muscle atrophy [28]. UCR has also been identified as a reliable marker for assessing skeletal muscle levels [29]. Notably, mCXI relies solely on serological markers, enhancing its practicality and suitability for routine clinical use. Malnutrition and systemic inflammation are well-documented factors influencing cancer prognosis [30]. In this cohort, patients with lower mCXI levels were more frequently diagnosed with malnutrition. Serum ALB, a critical marker of nutritional status in patients with gastrointestinal cancers [31], is closely linked to malnutrition when its levels are low [32]. Likewise, NLR, a marker of systemic inflammation, has been shown to significantly predict CRC prognosis [33, 34]. By combining ALB and NLR, mCXI provides a dual evaluation of nutritional status and inflammatory response, offering a more comprehensive prognostic tool. Multivariate Cox regression analysis consistently showed that patients with high mCXI levels had significantly better RFS and OS compared to those with low mCXI levels. These findings further underscore the prognostic value of mCXI and its potential clinical applicability.

In recent years, nomogram prediction models have gained significant traction in clinical practice due to their ability to provide individualized risk assessments. Unlike the traditional TNM staging system, nomogram models offer superior accuracy and surpass subjective clinical judgments in evaluating disease progression [35]. The nomogram model developed in this study based on mCXI, demonstrated excellent performance in predicting RFS and OS in elderly patients with CRC. Survival ROC curve analysis revealed AUC values of 0.809 for 3-year OS predictions and 0.798 for 3-year RFS predictions, highlighting the model's robust predictive ability. Meanwhile, we compared the AUC of the model with and without mCXI for predicting RFS and OS. The results showed that the model with mCXI predicted significantly better 3-year and 5-year RFS and OS than that of the model without mCXI, which indicated mCXI was a significant contribution to the predictive ability of the model. Calibration curves further confirmed the model's high accuracy, and DCA demonstrated its substantial net benefit in clinical decision making. The nomogram not only helps clinicians more accurately assess patient prognosis but also provides valuable guidance for formulating treatment strategies in elderly patients.

While this study introduced a novel tool for prognostic assessment in elderly patients with CRC, the study design presents several limitations. First, as a single-center retrospective study, the data may be prone to selection bias. Future multicenter, large-sample prospective studies are required to validate these findings. Additionally, while mCXI calculation is straightforward, its applicability across diverse populations warrants further exploration. Future research could investigate combining mCXI with other prognostic markers to improve model accuracy. Despite these limitations, this study was the first to establish a prognostic prediction model for elderly patients with CRC based on mCXI, offering a valuable reference for prognosis assessment.

## 5. Conclusion

The mCXI is an independent risk factor for both RFS and OS in elderly patients with CRC. The nomogram prediction model based on mCXI exhibits high accuracy and clinical utility in predicting patient prognosis, providing a novel tool for individualized treatment and prognostic assessment.

## Figures and Tables

**Table 1:** Comparison of clinical characteristics between the low mCXI and high mCXI groups.

Variables	Total (*N* = 456)	Low mCXI (≤0.63) (*N* = 289)	High mCXI (>0.63) (*N* = 167)	*p* Value
Age (years)	75.00	76.00	74.00	**0.023**
SMI	40.00	39.00	40.00	0.138
CXI	323.85	247.87	517.50	**<0.001**
Gender	—	—	—	0.236
Female	183 (40.1%)	110 (38.1%)	73 (43.7%)	—
Male	273 (59.9%)	179 (61.9%)	94 (56.3%)	—
Tumor size (cm)	—	—	—	0.582
≤5	304 (66.7%)	190 (65.7%)	114 (68.3%)	—
>5	152 (33.3%)	99 (34.3%)	53 (31.7%)	—
CEA	—	—	—	0.974
≤3.41	167 (36.6%)	106 (36.7%)	61 (36.5%)	—
>3.41	289 (63.4%)	183 (63.3%)	106 (63.5%)	—
*T*	—	—	—	0.372
1–2	109 (23.9%)	73 (25.3%)	36 (21.6%)	—
3–4	347 (76.1%)	216 (74.7%)	131 (78.4%)	—
*N*	—	—	—	0.203
0–1	374 (82.0%)	232 (80.3%)	142 (85.0%)	—
2	82 (18.0%)	57 (19.7%)	25 (15.0%)	—
Ki-67 (%)	—	—	—	**0.014**
≤75	258 (56.6%)	176 (60.9%)	82 (49.1%)	—
>75	198 (43.4%)	113 (39.1%)	85 (50.9%)	—
Nerve invasion	—	—	—	0.115
No	276 (60.5%)	167 (57.8%)	109 (65.3%)	—
Yes	180 (39.5%)	122 (42.2%)	58 (34.7%)	—
Vascular invasion	—	—	—	0.448
No	345 (75.7%)	222 (76.8%)	123 (73.7%)	—
Yes	111 (24.3%)	67 (23.2%)	44 (26.3%)	—
Neoadjuvant therapy	—	—	—	0.669
No	434 (95.2%)	276 (95.5%)	158 (94.6%)	—
Yes	22 (4.8%)	13 (4.5%)	9 (5.4%)	—

*Note:* Bold values indicate statistically significant differences between the high and low mCXI groups (*p* < 0.05).

Abbreviations: CXI, cachexia index; mCXI, modified cachexia index; SMI, skeletal muscle index.

**Table 2:** Univariate and multivariate Cox regression analysis for OS.

Variables	Comparison	Univariate analysis		Multivariate analysis	
		HR (95% CI)	*p* Value	HR (95% CI)	*p* Value
Gender	Male/female	0.963 (0.605, 1.532)	0.874	—	—
Age (years)	—	1.058 (1.015, 1.102)	**0.007**	1.054 (1.010, 1.100)	**0.017**
Size (cm)	>5/≤5	1.133 (0.703, 1.827)	0.607	—	—
CEA	—	1.001 (0.999, 1.003)	0.298		
*T*	3–4/1–2	3.799 (1.649, 8.755)	**0.002**	2.891 (1.232, 6.783)	**0.015**
*N*	2/0–1	5.325 (3.369, 8.416)	**<0.001**	4.249 (2.579, 7.000)	**<0.001**
Ki-67	>75%/≤75%	0.787 (0.493, 1.255)	0.314	—	—
Nerve invasion	Yes/no	1.526 (0.967, 2.407)	0.069	—	—
Vascular invasion	Yes/no	1.743 (1.081, 2.809)	**0.023**	1.078 (0.641, 1.813)	0.777
SMI	—	0.992 (0.964, 1.020)	0.571		
CXI	—	1.000 (0.999, 1.001)	0.381		
mCXI	—	0.104 (0.036, 0.300)	**<0.001**	0.124 (0.042, 0.359)	**<0.001**

*Note:* A *p* values < 0.05 indicates that the result is statistically significant. Statistically significant values are shown in bold.

Abbreviations: 95% CI, 95% confidence intervals; CXI, cachexia index; HR, hazard ratios; mCXI, modified cachexia index; SMI, skeletal muscle index.

**Table 3:** Univariate and multivariate Cox regression analysis for RFS.

Variables	Comparison	Univariate analysis		Multivariate analysis	
		HR (95% CI)	*p* Value	HR (95% CI)	*p* Value
Gender	Male/female	0.980 (0.616, 1.558)	0.931	—	—
Age (years)	—	1.056 (1.013, 1.100)	**0.010**	1.046 (1.002, 1.092)	**0.039**
Size (cm)	>5/≤5	1.146 (0.711, 1.848)	0.575	—	—
CEA	—	1.001 (0.999, 1.003)	0.318		
*T*	3–4/1–2	3.764 (1.633, 8.673)	**0.002**	2.943 (1.256, 6.896)	**0.013**
*N*	2/0–1	5.413 (3.424, 8.557)	**<0.001**	4.335 (2.627, 7.153)	**<0.001**
Ki-67	>75%/≤75%	0.796 (0.499, 1.269)	0.337	—	—
Nerve invasion	Yes/No	1.495 (0.947, 2.358)	0.084	—	—
Vascular invasion	Yes/No	1.724 (1.069, 2.779)	**0.025**	1.030 (0.612, 1.735)	0.910
SMI	—	0.993 (0.965, 1.022)	0.626		
CXI	—	1.000 (0.999, 1.001)	0.463		
mCXI	—	0.107 (0.037, 0.307)	**<0.001**	0.128 (0.045, 0.369)	**<0.001**

*Note:* A *p* value < 0.05 indicates that the result is statistically significant. Statistically significant values are shown in bold.

Abbreviations: 95% CI, 95% confidence intervals; CXI, cachexia index; HR, hazard ratios; mCXI, modified cachexia index; SMI, skeletal muscle index.

**Table 4:** Comparison of AUC in models with and without mCXI.

Survival	AUC with mCXI	AUC without mCXI	*p* Value (delong tset)
1-year RFS	0.769	0.735	0.085
3-year RFS	0.798	0.775	**0.019**
5-year RFS	0.797	0.745	**0.002**
1-year OS	0.742	0.721	0.28
3-year OS	0.809	0.794	**0.044**
5-year OS	0.799	0.741	**0.001**

*Note:* A *p* value < 0.05 indicates that the result is statistically significant. Statistically significant values are shown in bold.

## Data Availability

The datasets generated and analyzed during the current study are available from the corresponding author upon reasonable request. The data are not publicly available due to privacy or ethical restrictions.

## Supporting Information

Supporting Information 1 Figure S1: The AUC of nomogram model. (A) 1-, 3-, and 5-year OS (with CXI) prediction. (B) 1-, 3-, and 5-year RFS (with CXI) prediction.

Supporting Information 2 Table SI: The comparison of AUC in three models.

## References

[1] Sung H., Ferlay J., Siegel R. L. (2021). Global Cancer Statistics 2020: GLOBOCAN Estimates of Incidence and Mortality Worldwide for 36 Cancers in 185 Countries. *CA: A Cancer Journal for Clinicians*.

[2] Wang Z., Dan W., Zhang N., Fang J., Yang Y. (2023). Colorectal Cancer and Gut Microbiota Studies in China. *Gut Microbes*.

[3] Elliot M. S., Todd I. P., Nicholls R. J. (1982). Radical Restorative Surgery for Poorly Differentiated Carcinoma of the Mid-Rectum. *Journal of British Surgery*.

[4] Shalata W., Gluzman A., Man S. (2024). Colorectal Cancer in Elderly Patients: Insights into Presentations, Prognosis, and Patient Outcomes. *Medicina (Kaunas, Lithuania)*.

[5] Faivre J., Lemmens V. E., Quipourt V., Bouvier A. M. (2007). Management and Survival of Colorectal Cancer in the Elderly in Population-Based Studies. *European Journal of Cancer*.

[6] Hayama T., Hashiguchi Y., Ozawa T. (2022). The Preoperative Geriatric Nutritional Risk Index (GNRI) Is an Independent Prognostic Factor in Elderly Patients Underwent Curative Resection for Colorectal Cancer. *Scientific Reports*.

[7] Keskinkilic M., Semiz H. S., Ataca E., Yavuzsen T. (2024). The Prognostic Value of Immune-Nutritional Status in Metastatic Colorectal Cancer: Prognostic Nutritional Index (PNI). *Supportive Care in Cancer*.

[8] Xie H., Gong Y., Kuang J. (2020). Computed Tomography-Determined Sarcopenia is a Useful Imaging Biomarker for Predicting Postoperative Outcomes in Elderly Colorectal Cancer Patients. *Cancer Research and Treatment*.

[9] Go S.-I., Park M. J., Lee G.-W. (2021). Clinical Significance of the Cachexia Index in Patients With Small Cell Lung Cancer. *BMC Cancer*.

[10] Okubo S., Shinmura K., Kadota S. (2024). Evaluation of the Cachexia Index Using a Bioelectrical Impedance Analysis in Elderly Patients With Non-Hodgkin’s Lymphoma: A Single-Center Prospective Study. *Annals of Hematology*.

[11] Yuan Q., Liu L., Wang K. (2024). Developing and Validating a Modified Cachexia Index to Predict the Outcomes for Colorectal Cancer After Radical Surgery. *European Journal of Clinical Nutrition*.

[12] Kim Y. Y., Lee J., Jeong W. K. (2021). Prognostic Significance of Sarcopenia in Microsatellite-Stable Gastric Cancer Patients Treated With Programmed Death-1 Inhibitors. *Gastric Cancer*.

[13] Oke S. M., Rye B., Malietzis G. (2020). Survival and CT Defined Sarcopenia in Patients With Intestinal Failure on Home Parenteral Support. *Clinical Nutrition*.

[14] McKeown E., Nelson D. W., Johnson E. K. (2014). Current Approaches and Challenges for Monitoring Treatment Response in Colon and Rectal Cancer. *Journal of Cancer*.

[15] Urruticoechea A., Smith I. E., Dowsett M. (2005). Proliferation Marker Ki-67 in Early Breast Cancer. *Journal of Clinical Oncology*.

[16] Wan Q., Yuan Q., Zhao R. (2022). Prognostic Value of Cachexia Index in Patients With Colorectal Cancer: A Retrospective Study. *Frontiers in Oncology*.

[17] Rinninella E., Cintoni M., Raoul P. (2020). Muscle Mass, Assessed at Diagnosis by L3-CT Scan as a Prognostic Marker of Clinical Outcomes in Patients With Gastric Cancer: A Systematic Review and Meta-Analysis. *Clinical Nutrition*.

[18] Pamoukdjian F., Bouillet T., Lévy V., Soussan M., Zelek L., Paillaud E. (2018). Prevalence and Predictive Value of Pre-Therapeutic Sarcopenia in Cancer Patients: A Systematic Review. *Clinical Nutrition*.

[19] Xia L., Zhao R., Wan Q. (2020). Sarcopenia and Adverse Health-Related Outcomes: An Umbrella Review of Meta-Analyses of Observational Studies. *Cancer Medicine*.

[20] Xiao J., Caan B. J., Cespedes Feliciano E. M. (2020). Association of Low Muscle Mass and Low Muscle Radiodensity With Morbidity and Mortality for Colon Cancer Surgery. *JAMA Surgery*.

[21] Olmez T., Karakose E., Bozkurt H. (2021). Sarcopenia Is Associated With Increased Severe Postoperative Complications After Colon Cancer Surgery. *Archives of Medical Science*.

[22] Cruz-Jentoft A. J., Bahat G., Bauer J. (2019). Sarcopenia in Older People 2 (EWGSOP2), and the Extended Group for EWGSOP2. *Age and Ageing*.

[23] Mourtzakis M., Prado C. M., Lieffers J. R., Reiman T., McCargar L. J., Baracos V. E. (2008). A Practical and Precise Approach to Quantification of Body Composition in Cancer Patients Using Computed Tomography Images Acquired During Routine Care. *Applied Physiology, Nutrition, and Metabolism*.

[24] Argilés J. M., Busquets S., Stemmler B., López-Soriano F. J. (2014). Cancer Cachexia: Understanding the Molecular Basis. *Nature Reviews Cancer*.

[25] Haines R. W., Zolfaghari P., Wan Y., Pearse R. M., Puthucheary Z., Prowle J. R. (2019). Elevated Urea-to-Creatinine Ratio Provides a Biochemical Signature of Muscle Catabolism and Persistent Critical Illness After Major Trauma. *Intensive Care Medicine*.

[26] Puthucheary Z. A., Astin R., Mcphail M. J. W. (2018). Metabolic Phenotype of Skeletal Muscle in Early Critical Illness. *Thorax*.

[27] Gamrin-Gripenberg L., Sundström-Rehal M., Olsson D., Grip J., Wernerman J., Rooyackers O. (2018). An Attenuated Rate of Leg Muscle Protein Depletion and Leg Free Amino Acid Efflux Over Time Is Seen in ICU Long-Stayers. *Critical Care*.

[28] Herridge M. S., Cheung A. M., Tansey C. M. (2003). One-Year Outcomes in Survivors of the Acute Respiratory Distress Syndrome. *New England Journal of Medicine*.

[29] Gao H., Wang J., Zou X., Zhang K., Zhou J., Chen M. (2022). High Blood Urea Nitrogen to Creatinine Ratio Is Associated With Increased Risk of Sarcopenia in Patients With Chronic Obstructive Pulmonary Disease. *Experimental Gerontology*.

[30] Lu J. N., Zhou L. S., Zhang S., Li J. X., Xu C. J. (2024). Performance of Nutritional and Inflammatory Markers in Patients With Pancreatic Cancer. *World Journal of Clinical Oncology*.

[31] Gupta A., Gupta E., Hilsden R. (2021). Preoperative Malnutrition in Patients With Colorectal Cancer. *Canadian Journal of Surgery*.

[32] Yamamoto T., Kawada K., Obama K. (2021). Inflammation-Related Biomarkers for the Prediction of Prognosis in Colorectal Cancer Patients. *International Journal of Molecular Sciences*.

[33] Ming-Sheng F., Mei-Ling D., Xun-Quan C., Yuan-Xin H., Wei-Jie Z., Qin-Cong P. (2022). Preoperative Neutrophil-to-Lymphocyte Ratio, Platelet-to-Lymphocyte Ratio, and CEA as the Potential Prognostic Biomarkers for Colorectal Cancer. *Canadian Journal of Gastroenterology and Hepatology*.

[34] Schiefer S., Wirsik N. M., Kalkum E. (2022). Systematic Review of Prognostic Role of Blood Cell Ratios in Patients With Gastric Cancer Undergoing Surgery. *Diagnostics*.

[35] Balachandran V. P., Gonen M., Smith J. J., DeMatteo R. P. (2015). Nomograms in Oncology: More Than Meets the Eye. *The Lancet Oncology*.

