# Supplementary figures and images for: Impact of New Inflammation/Nutrition-Based Indicators on Prognosis in Elderly Patients With Colorectal Cancer
Source: Mediators Inflamm. 2025 Nov 19;2025:7843467. doi: 10.1155/mi/7843467 (PMC12657088; doi:10.1155/mi/7843467)

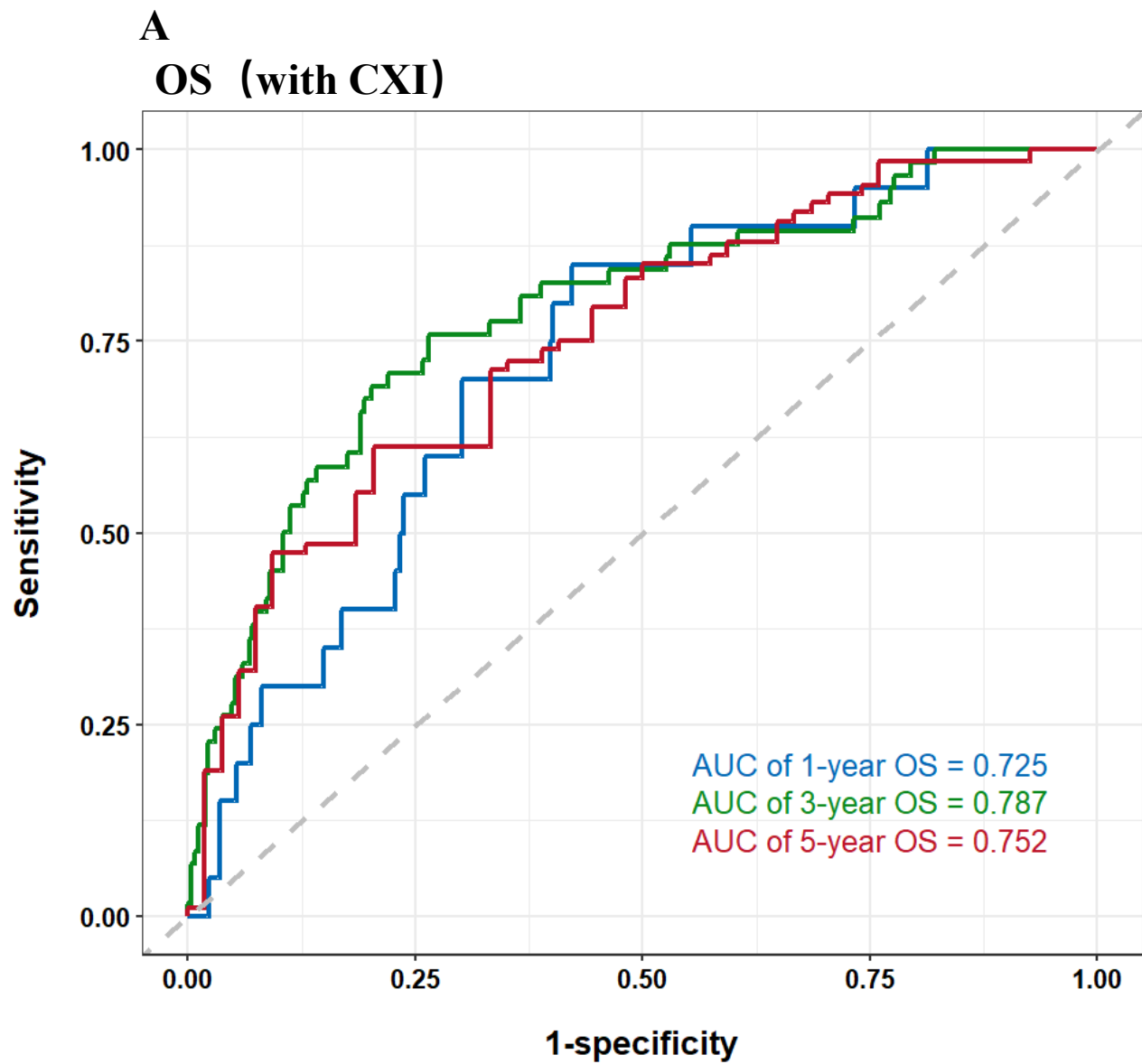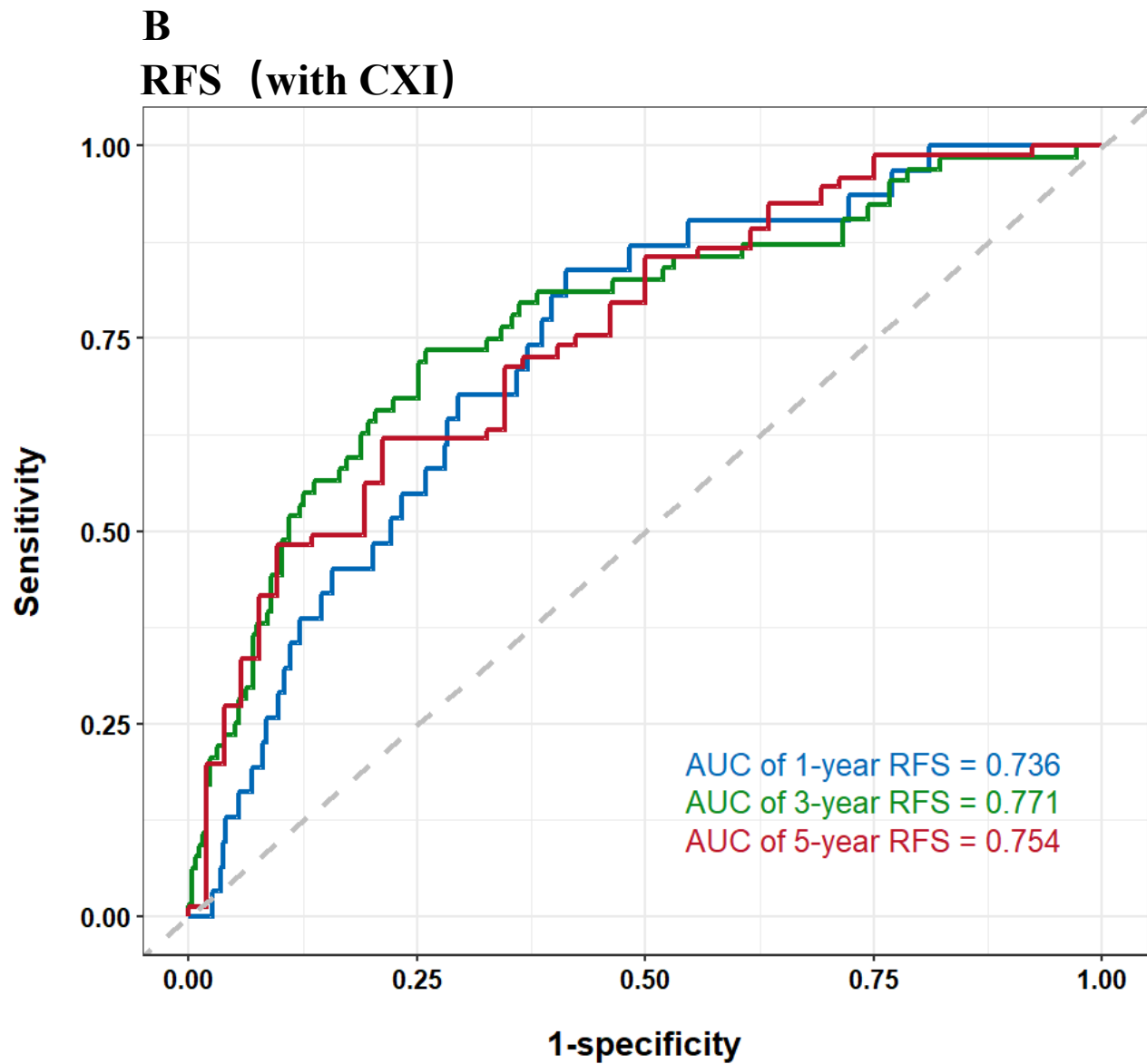

**Supplementary Figure 1**

Supplement: Supporting Information 1 — Figure S1: The AUC of nomogram model. (A) 1-, 3-, and 5-year OS (with CXI) prediction. (B) 1-, 3-, and 5-year RFS (with CXI) prediction. [file 7843467.f1.pdf]
