# Supplementary material for: Impact of New Inflammation/Nutrition-Based Indicators on Prognosis in Elderly Patients With Colorectal Cancer
Source: Mediators Inflamm. 2025 Nov 19;2025:7843467. doi: 10.1155/mi/7843467 (PMC12657088; doi:10.1155/mi/7843467)
Supplement: Supporting Information 2 — Table SI: The comparison of AUC in three models. [file 7843467.f2.docx]

**Supplementary table 1. The comparison of AUC in three models**

| Survival | AUC with mCXI | AUC without mCXI | AUC with CXI* |
| --- | --- | --- | --- |
| 1-year RFS | 0.769 | 0.735 | 0.736 |
| 3-year RFS | 0.798 | 0.775 | 0.771 |
| 5-year RFS | 0.797 | 0.745 | 0.754 |
| 1-year OS | 0.742 | 0.721 | 0.725 |
| 3-year OS | 0.809 | 0.794 | 0.787 |
| 5-year OS | 0.799 | 0.741 | 0.752 |

*The model containing CXI includes age, T stage, N stage, and CXI, and does not include mCXI.
